# Development and internal validation of a risk stratification model for high-intensity psychosocial service use among women living with and beyond breast cancer

**DOI:** 10.1186/s12905-026-04726-2

**Published:** 2026-07-30

**Authors:** Limor Dina Gonen, Sharon Barak, Riki Tesler, Avi Zigdon

**Affiliations:** 1 Department of Economics and Business Administration, Ariel University, P.O.B. 3, Kiryat HaMada, Ariel, 40700 Israel; 2 Department of Nursing, School of Health Sciences, Ariel University, Ariel, Israel; 3 Department of Health Systems Management, School of Health Sciences, Ariel University, Ariel, Israel

**Keywords:** Breast Neoplasms, Cancer Survivors, Psychological Distress, Psychosocial Support Systems, Health Services Accessibility, Socioeconomic Factors, Patient-Reported Outcome Measures, Health Equity

## Abstract

**Purpose:**

Breast cancer survivorship is frequently accompanied by persistent psychosocial distress and heterogeneous supportive care needs. Although distress screening is widely recommended, limited evidence exists on pragmatic tools to identify survivors most likely to require high-intensity psychosocial engagement. Because breast cancer survivorship is a major women’s health issue, tools that support more equitable and timely identification of women with greater supportive care needs may have direct relevance for women-centered service planning. We aimed to develop and internally validate a risk stratification model for high-intensity psychosocial service use and assess the incremental value of a distress index.

**Methods:**

We conducted a cross-sectional risk stratification study among 411 women living with or after breast cancer who completed an anonymous online self-report questionnaire disseminated through community-based and professional recruitment channels in Israel. High-intensity service use was defined as the upper tertile of cumulative engagement. A base logistic regression model included demographic and clinical predictors; an expanded model added a composite distress index. Discrimination and calibration were evaluated using stratified 5-fold cross-validation and bootstrap internal validation.

**Results:**

The base model showed moderate discrimination (cross-validated AUC = 0.675; bootstrap median AUC = 0.706, 95% CI 0.687–0.715). Adding the distress index improved discrimination (cross-validated AUC = 0.748; bootstrap median AUC = 0.778, 95% CI 0.763–0.785) and reduced model error, with the Brier score improving from 0.196 to 0.180. Lower income and shorter time since diagnosis were independently associated with high psychosocial service use.

**Conclusions:**

A pragmatic risk stratification model demonstrated moderate ability to identify survivors likely to engage intensively with psychosocial services. Incorporating a brief distress index meaningfully enhanced model discrimination and classification performance.

**Implications for cancer survivors:**

Risk-based targeting increased positive predictive value to 0.60–0.69 in high-risk strata, suggesting that proactive outreach based on predicted risk could more efficiently concentrate psychosocial resources among survivors most likely to require sustained support. These findings may also inform women’s health programs seeking to improve supportive care access for women facing psychosocial or socioeconomic vulnerability after breast cancer.

**Supplementary Information:**

The online version contains supplementary material available at https://doi.org/10.1186/s12905-026-04726-2.

## Introduction

Improvements in early detection and systemic therapies have substantially increased survival following breast cancer, resulting in a growing population of long-term survivors worldwide [1, 2]. As survival improves, attention has shifted from acute treatment outcomes to survivorship care, encompassing physical functioning, psychological well-being, and social reintegration [3, 4]. Survivorship is now recognized as a distinct phase of the cancer continuum requiring structured and coordinated care, extending beyond surveillance for recurrence to include symptom management, psychosocial well-being, care coordination, and long-term follow-up needs [5, 6]. Because breast cancer predominantly affects women, survivorship care in this context is also a central women’s health concern, particularly where psychosocial burden, family and caregiving responsibilities, role functioning, employment disruption, financial strain, and access to supportive care intersect.

Psychological distress remains one of the most prevalent and persistent challenges in breast cancer survivorship. Recent meta-analytic evidence indicates that approximately 38% of women with breast cancer experience clinically significant psychological distress [7]. Furthermore, broader evidence across cancer populations suggests that depression and anxiety affect approximately 33.2% and 30.6% of cancer survivors, respectively [8]. Symptoms of anxiety and depression frequently extend beyond the completion of active treatment. Fear of cancer recurrence remains one of the most common and enduring survivorship concerns and may remain clinically significant for years after treatment completion [9–11]. Contemporary evidence continues to document substantial unmet psychosocial needs among breast cancer survivors, particularly within emotional and psychological domains [12, 13]. Persistent psychological distress has been consistently associated with diminished health-related quality of life, impaired functional recovery, and greater healthcare utilization [14, 15]. Among women living with and beyond breast cancer, these effects may be further compounded by caregiving responsibilities, financial strain, and broader gendered social expectations, highlighting the importance of timely identification of supportive care needs.

Clinical guidelines issued by the American Cancer Society/American Society of Clinical Oncology (ACS/ASCO) and the National Comprehensive Cancer Network (NCCN) recommend routine assessment of psychological distress and referral to appropriate psychosocial support services throughout survivorship care [3, 16].

However, screening alone does not necessarily translate into effective service allocation, as identification of distress does not guarantee subsequent referral, access to, or engagement with appropriate psychosocial support services [16, 17]. Distress severity identifies symptom burden but does not inherently distinguish survivors who will require sustained, high-intensity psychosocial engagement from those whose needs may resolve with minimal support. Moreover, psychosocial service use is influenced by socioeconomic context, time since diagnosis, and disease characteristics [12, 18].

Community-based psychosocial programs play a critical role in survivorship care, particularly in health systems where supportive services are delivered outside hospital oncology settings. Such programs often operate with finite staffing and budgetary resources, creating a need for efficient targeting strategies. While structured risk stratification models are increasingly used in oncology to guide surveillance and care coordination [19], comparable models to identify intensive psychosocial service use remain underdeveloped.

Most existing survivorship research describes prevalence of distress or unmet need rather than developing pragmatic tools for prediction and triage. Systematic reviews highlight the heterogeneity of survivors’ supportive care needs but note the absence of validated models to identify individuals most likely to require intensive psychosocial intervention [12, 14]. From both health-services and women’s-health perspectives, the absence of risk-based targeting tools may result in inefficient allocation of psychosocial resources and missed opportunities to identify survivors experiencing heightened psychosocial and socioeconomic vulnerability.

Risk stratification modelling provides a structured methodological framework for estimating individualized risk without assuming causality, and for informing resource allocation decisions in clinical and community settings [20, 21]. Within survivorship care, a validated risk stratification approach could support proactive identification of survivors at elevated likelihood of high-intensity service engagement. Furthermore, although distress screening is widely implemented, the incremental value of incorporating a brief distress index into a demographic and clinical risk stratification model remains uncertain.

Against this background, the present study aimed to develop and internally validate a risk stratification model for high-intensity psychosocial service use among breast cancer survivors engaged in community-based support services. Specifically, we sought to evaluate the discrimination and calibration performance of a demographic and clinical risk stratification model, assess the incremental classification value of a composite distress index, and examine the potential utility of risk-based targeting strategies for concentrating psychosocial outreach resources.

By focusing on pragmatic risk stratification within a survivorship support framework, this study addresses how breast cancer survivors most likely to require intensive psychosocial engagement can be identified more efficiently when supportive-care resources are limited.

## Methods

### Study design and setting

This cross-sectional analytic study aimed to develop and internally validate a risk stratification model for high-intensity psychosocial support use among women with a history of breast cancer. The analytic objective was risk stratification and concurrent classification rather than causal inference or prognostic prediction, with the goal of informing targeted survivorship outreach strategies within a publicly financed healthcare system. Given the growing emphasis on comprehensive women-centered survivorship care, the study was also intended to inform practical approaches to identifying women who may benefit most from intensive psychosocial support.

Data were collected in 2024 using a self-administered, anonymous web-based questionnaire. All study variables were obtained through participant self-report. No data were extracted from medical records, hospital databases, clinical registries, organizational records, or other clinician-reported sources. The survey was disseminated through multiple recruitment channels, including the “One in Nine” nationwide breast cancer patient organization in Israel, Facebook, social media platforms, email, WhatsApp groups, and oncology healthcare professionals who shared the survey with potentially eligible women. The “One in Nine” organization provides psychosocial support services outside formal oncology settings, including counseling, peer support, and group-based interventions. All demographic, clinical, comorbidity, psychosocial distress, and psychosocial service-use variables were self-reported by participants.

Participation was entirely voluntary. Before beginning the questionnaire, participants were presented with an informed consent statement and were required to actively indicate their agreement to participate. Participants were informed that they could discontinue participation at any time prior to questionnaire submission without penalty or consequence. No financial, material, or other incentives were offered for participation.

Model development and reporting were guided by the TRIPOD (Transparent Reporting of a multivariable prediction model for Individual Prognosis or Diagnosis) recommendations for multivariable prediction and classification model studies [20].

### Participants

**Fig. 1:**
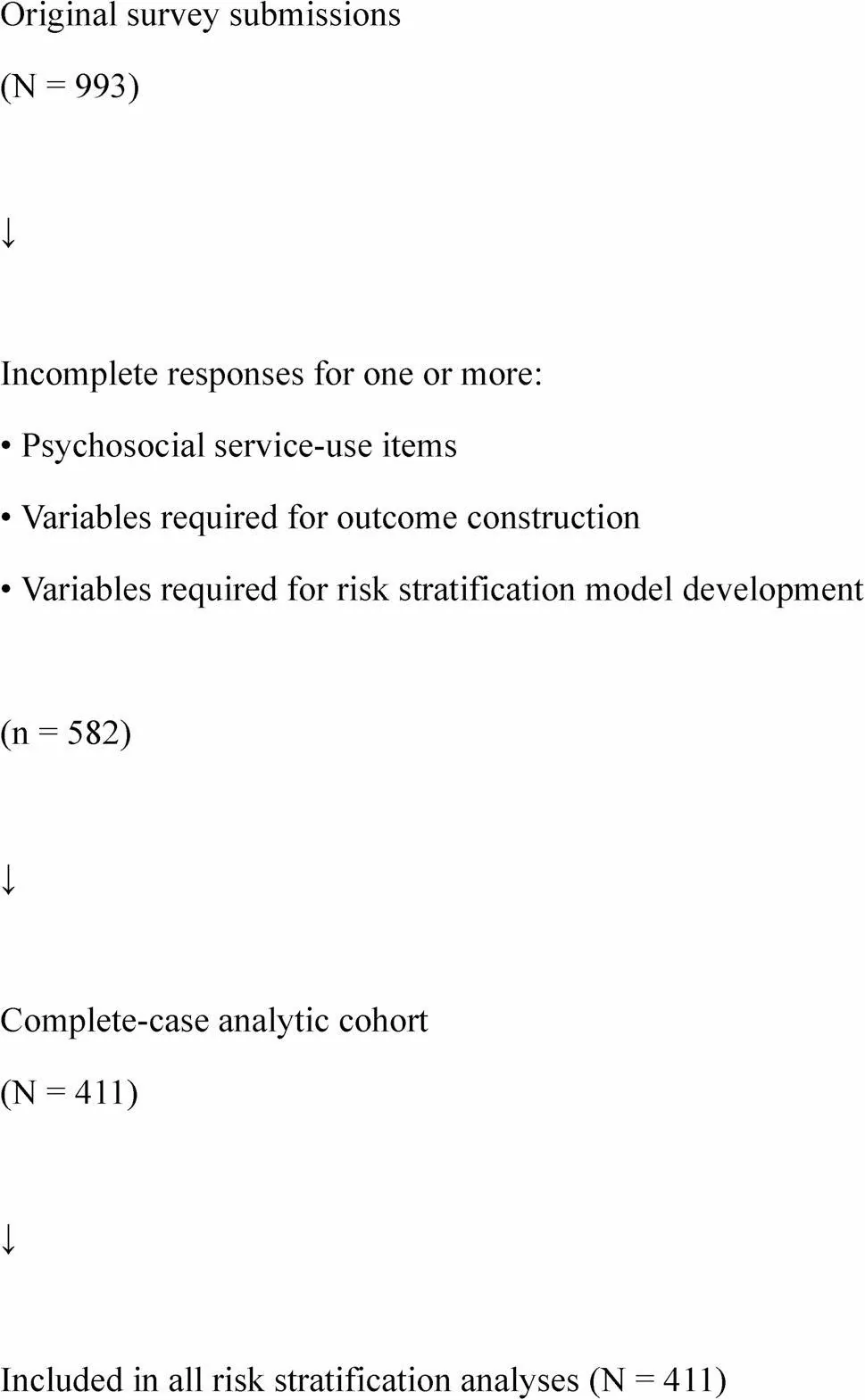
Participant Flow Diagram for Construction of the Analytic Cohort. Participant flow diagram describing derivation of the final analytic cohort used for model development and internal validation. The diagram summarizes the transition from the original survey dataset to the final complete-case analytic cohort used in all risk stratification analyses. The original survey dataset contained 993 survey submissions. The final analytic cohort consisted of 411 women meeting all predefined complete-case inclusion criteria. Exclusions primarily reflected incomplete questionnaire completion or missing information required for outcome construction or risk stratification model development. No imputation procedures were used in constructing the analytic cohort

The derivation of the final analytic cohort is summarized in Fig. 1.

The final analytic sample consisted of women who completed all psychosocial service-use items and all variables required for risk stratification model development. Consequently, the analytic cohort represents a complete-case sample derived from a lengthy modular survivorship survey rather than exclusions based on outcome status or model predictions.

The reduction from 993 survey submissions to 411 analytic observations primarily reflected incomplete questionnaire completion and missing data in variables required for outcome construction and risk stratification model development. A total of 582 submissions were excluded because of incomplete questionnaire completion or missing information required for outcome construction or risk stratification model development.

Missing survey responses were not imputed for cohort construction or participant inclusion purposes.

Participants were adult women with a current or previous diagnosis of breast cancer, representing varying disease stages and time intervals since diagnosis. Eligibility criteria required participants to be women aged 18 years or older, residing in Israel, Hebrew-speaking, and currently living with or recovering from breast cancer. Because the survey was disseminated through multiple community-based, online, and professional recruitment channels, the total number of women exposed to the survey invitation could not be determined; therefore, a formal response rate could not be calculated.

In addition, aggregate demographic and clinical information describing the full population served by the support organization was not available to the research team. Consequently, formal assessment of sample representativeness relative to the broader population served by the organization was not possible.

All data were de-identified prior to analysis.

### Outcome definition

The primary outcome was high-intensity psychosocial service use.

Participants were categorized into tertiles based on a cumulative psychosocial service use score (range 0–27). The cumulative score was derived from 27 psychosocial support service domains included in the structured survey administered by the survivorship support organization. For each domain, responses indicating current or previous engagement with the service (“Yes” or “Mainly now”) were coded as 1, whereas responses indicating no use (“No”) were coded as 0. The 27 binary indicators were summed to create a cumulative psychosocial service-use breadth score ranging from 0 to 27, with higher scores indicating engagement with a broader range of psychosocial support services. The resulting score reflects the breadth of psychosocial service engagement across support domains rather than the frequency or intensity of use within any individual service.

Participants were categorized using tertile-based cut-points derived from the observed distribution of the cumulative score: low use (0–9), moderate use (10–16), and high use (17–27). For the primary risk stratification analysis, the high-use group was coded as 1, whereas the low- and moderate-use groups (0–16) were combined and coded as 0 (“lower use”).

This binary operationalization reflects a service-targeting framework intended to identify survivors most likely to require intensive psychosocial engagement. The outcome should therefore be interpreted as a measure of breadth of psychosocial service engagement within the support program rather than as a clinically validated measure of psychosocial need. The tertile-based definition was selected to reflect relative service-use intensity within this program rather than a pre-specified clinical cut-off. Because no standardized threshold for high-intensity psychosocial engagement exists in community-based survivorship settings, a distribution-based approach was used to operationalize the risk-stratification outcome. Baseline distribution of outcome groups is presented in Table 1.

To evaluate the potential information loss associated with dichotomization, supplementary analyses examined the distribution of the original cumulative psychosocial service-use score and its association with model-predicted risk. These analyses are presented in Supplementary Table S1.

### Predictor variables

Predictors were selected a priori based on clinical plausibility, feasibility of collection in survivorship settings, and potential relevance to service demand. Additional psychosocial and family-context variables were evaluated during model development; however, only predictors demonstrating meaningful incremental contribution to discrimination, overall model performance, and practical applicability were retained in the final parsimonious model. All predictor variables were obtained from the anonymous online self-report questionnaire. Clinical variables, including disease stage, metastatic status, and years since breast cancer diagnosis, were reported directly by participants and were not verified against medical records.

### Demographic and clinical variables

The base (parsimonious) model included age (continuous), years since breast cancer diagnosis (continuous), employment status (employed or self-employed vs. not employed), household income (below average, average, above average [reference], or prefer not to answer), presence of at least one comorbidity (yes/no), and disease stage categorized as early stage (DCIS–III; reference), metastatic disease, or survivor/unknown stage.

Years since diagnosis was derived from the self-reported year of initial breast cancer diagnosis and was expressed in whole years relative to the survey year (2024). Month of diagnosis was not collected and therefore months since diagnosis were not available. Disease stage categories were grouped to ensure adequate cell sizes and maintain clinical interpretability.

Household income was self-reported using relative income categories referenced to the national average monthly gross wage in Israel, which was presented in the survey instrument as 12,860 NIS per month. This reference value corresponded to the average monthly wage of salaried employees in Israel in 2023 [22]. Participants classified their household income as much below average, below average, around average, above average, or much above average. Exact household income amounts were not collected.

Comorbidity status was based on self-report. Participants were presented with a list of chronic health conditions, including cardiovascular, pulmonary, metabolic, neurologic, autoimmune, psychiatric, renal, hepatic, and other chronic diseases. Participants reporting at least one chronic condition in addition to breast cancer were classified as having ≥ 1 comorbidity, whereas those reporting no additional chronic conditions were classified as having none. Physician verification was not available because no medical records were used. Because comorbidities were not verified against medical records and physician diagnosis was not confirmed in the present study, this variable should be interpreted as self-reported comorbidity burden.

Baseline characteristics stratified by service-use group are presented in Table 1.

### Expanded model: distress burden

To evaluate whether brief symptom screening improves classification performance, an expanded model additionally included a composite distress index. This index was calculated as the respondent-level mean across seven psychological symptom items assessing anxiety, low mood, emotional distress, and related psychosocial symptom domains, each scored on a 0–3 scale (0 = not at all, 1 = several days, 2 = more than half the days, 3 = nearly every day), with higher values indicating greater distress burden.

This construct was considered particularly relevant in a women’s health context, where psychosocial symptom burden may influence not only emotional well-being but also daily functioning, family roles, and engagement with supportive services. The items were derived from a structured psychosocial survey instrument administered by the organization. The complete wording of the seven survey items and response options used to construct the composite distress index is provided in Supplementary Appendix A. Although not based on a single standardized diagnostic scale (e.g., HADS or PHQ-9), the items reflect commonly assessed domains of anxiety, mood disturbance, and emotional burden in survivorship research. The mean score was used rather than a summed score to preserve the original 0–3 scaling and facilitate interpretability across items. Internal consistency of the composite index in this sample was excellent (Cronbach’s α = 0.92). Corrected item–total correlations ranged from 0.69 to 0.83, and alpha coefficients remained stable when individual items were removed (range 0.90–0.91), supporting internal coherence of the construct. A basic dimensionality check indicated a dominant first factor (eigenvalue = 4.73), consistent with a largely unidimensional structure. The distress index was treated as a continuous predictor and evaluated for incremental contribution to model discrimination and classification performance, beyond demographic and clinical factors.

### Statistical analysis

#### Model specification

Multivariable logistic regression was used to estimate predicted probabilities of high-intensity psychosocial service use.

Because the objective was risk stratification and classification rather than causal inference, model performance metrics were emphasized over hypothesis testing. To reduce overfitting and improve stability, ridge-penalized logistic regression (L2 regularization) was implemented within a preprocessing pipeline that included median imputation and standardization for numeric predictors, as well as mode imputation and one-hot encoding for categorical predictors. Missing predictor values were handled within the preprocessing pipeline using median imputation for numeric variables and most-frequent-category imputation for categorical variables. This approach was selected because the primary objective of the study was risk stratification and classification rather than causal inference, and because the extent of missingness among model predictors was limited. Simple imputation procedures are commonly used in internally validated risk stratification models because they provide a transparent, computationally stable, and reproducible approach to handling limited missing predictor information. More complex approaches such as Multiple Imputation by Chained Equations (MICE) were therefore not considered necessary in the present setting [21].

Regularization methods are recommended for improving generalizability and reducing coefficient instability in multivariable risk stratification models, particularly in moderate sample sizes [21].

The analytic sample included 128 events (participants with high-intensity psychosocial service use). The expanded model incorporated nine predictors (including categorical levels), yielding an events-per-variable (EPV) ratio of approximately 14. This EPV exceeds commonly cited minimum thresholds for logistic regression model stability [23]. However, contemporary methodological guidance emphasizes that required sample size depends on anticipated model performance, predictor distribution, and overfitting risk rather than on a single universal EPV rule [24]. In addition, ridge penalization and internal validation procedures were applied to further enhance model robustness and mitigate small-sample bias.

For interpretability, conventional (unpenalized) logistic regression models were additionally estimated to present odds ratios (ORs) with 95% confidence intervals (Table 2).

### Internal validation

Model discrimination was evaluated using stratified 5-fold cross-validation to obtain internally validated performance estimates. In each fold, predicted probabilities were generated for participants not included in model fitting (out-of-fold predictions), thereby reducing optimism bias and preserving independence between model development and evaluation. Discrimination was quantified using the area under the receiver operating characteristic curve (AUC), representing the probability that the model assigns a higher predicted risk to a randomly selected participant with high-intensity psychosocial service use than to a participant with lower psychosocial service use [25].

The use of cross-validated predicted probabilities aligns with contemporary methodological guidance for risk stratification and classification modelling, which discourages simple data splitting and emphasizes robust internal validation procedures to limit overoptimistic performance estimates [26]. Model development and reporting adhered to the TRIPOD recommendations for transparent reporting of multivariable prediction models [20].

Calibration was assessed using calibration plots across deciles of predicted risk (Fig. 3 for the expanded model; Supplementary Figure S2 for the base model) and bootstrap internal validation with 500 resamples to estimate optimism-corrected AUC, calibration slope, and calibration intercept.

Bootstrap validation provides robust internal performance assessment and is widely recommended for risk stratification model development [27].

Overall model performance was summarized using the Brier score.

Detailed validation results and threshold-specific performance metrics are provided in the Supplementary Material (Supplementary Figures S1–S2 and Supplementary Table S2).

### Risk-based triage evaluation

To assess practical applicability, predicted probabilities were used to simulate targeted outreach strategies based on the top 20% and top 10% of predicted risk. For each threshold, sensitivity, specificity, positive predictive value (PPV), and negative predictive value (NPV) were calculated. These metrics quantify the efficiency of concentrating psychosocial outreach resources among survivors most likely to require high-intensity support.

In the context of women’s health service delivery, such targeting may be especially relevant where psychosocial resources are limited and where women with greater socioeconomic vulnerability may otherwise remain underserved. Detailed performance metrics are presented in Supplementary Table S2.

### Software

All statistical analyses were conducted using Python (version 3.11). Logistic regression models were implemented using standard statistical and machine learning libraries (scikit-learn and statsmodels).

### Ethical Considerations

This study received ethics approval from the Ariel University Ethics Committee (Approval No. AU-HEA-AZ-20240610). Before starting the anonymous online questionnaire, participants received an informed consent statement and were required to actively indicate their agreement to participate. Participation was voluntary, and participants were informed that they could discontinue participation at any time before submitting the questionnaire, without consequence. The questionnaire was completely anonymous, and no personally identifiable information was collected. No financial or other incentives were offered for participation. All study procedures complied with the Declaration of Helsinki.

## Results

### Participant characteristics

The analytic cohort included 411 breast cancer survivors. Of these, 128 (31.1%) were classified as participants with high psychosocial service use and 283 (68.9%) as participants with lower psychosocial service use.

Baseline demographic and clinical characteristics of women living with breast cancer, stratified by service-use group, are presented in Table 1. Mean age was comparable between groups. Participants in the high psychosocial service-use group had fewer years since diagnosis on average and were more likely to report lower household income. The prevalence of at least one comorbidity was modestly higher among participants in the high psychosocial service use group. Distribution of disease stage did not substantially differ between groups. These descriptive patterns suggest that psychosocial support intensity among women living with breast cancer may be shaped not only by clinical factors but also by social and economic vulnerability.

**Table 1:** Baseline characteristics of women living with breast cancer by psychosocial service use group

Characteristic	High use (*n* = 128)	Lower use (*n* = 283)
Age, mean (SD)	51.0 (10.3)	50.9 (10.5)
Years since diagnosis, mean (SD)	2.9 (3.1)	3.6 (3.2)
Employed, n (%)	48 (37.5)	146 (51.6)
≥ 1 comorbidity, n (%)	45 (35.2)	90 (31.8)
Income – Below average, n (%)	57 (44.5)	62 (21.9)
Income – Average, n (%)	33 (25.8)	87 (30.7)
Income – Above average, n (%)	24 (18.8)	117 (41.3)
Income – Prefer not to answer, n (%)	14 (10.9)	17 (6.0)
Stage – Early/DCIS–III, n (%)	51 (39.8)	69 (24.4)
Stage – Metastatic, n (%)	7 (5.5)	19 (6.7)
Stage – Survivor/unknown, n (%)	70 (54.7)	195 (68.9)

Baseline demographic, socioeconomic, clinical, and health-related characteristics of women with breast cancer according to psychosocial service-use group

Participant characteristics are presented separately for women classified as having high psychosocial service use and those classified as having lower psychosocial service use

Values are presented as mean (standard deviation, SD) for continuous variables and n (%) for categorical variables. High psychosocial service use was defined as the upper tertile (17–27) of the cumulative psychosocial service-use score. Lower psychosocial service use included participants in the low- and moderate-use groups (score 0–16). Income categories were self-reported household income categories referenced to the national average monthly wage presented in the survey instrument. Disease stage was self-reported. The category “Early/DCIS–III” includes ductal carcinoma in situ (DCIS) and non-metastatic breast cancer stages I–III. The category “Metastatic” corresponds to Stage IV disease. The category “Survivor/unknown stage” includes participants who identified themselves as breast cancer survivors but did not report a specific disease stage. Percentages may not total 100% because of rounding.

### Risk stratification model performance

#### Base model (demographic and clinical predictors)

The base model demonstrated moderate discrimination. Using 5-fold cross-validation, the area under the receiver operating characteristic curve (AUC) was 0.675. Bootstrap internal validation yielded a median AUC of 0.706 (95% CI: 0.687–0.715). The Brier score was 0.196, indicating moderate overall classification accuracy.

Discrimination and calibration of the base model are presented in Supplementary Figures S1 and S2.

Calibration plots demonstrated reasonable agreement between predicted and observed probabilities across deciles of risk.

#### Expanded model including distress burden

The addition of the distress index meaningfully improved model performance. The expanded model achieved a cross-validated AUC of 0.748 and a bootstrap median AUC of 0.778 (95% CI: 0.763–0.785). The Brier score improved to 0.180, indicating better overall classification performance compared with the base model.

The improvement in AUC indicates enhanced discrimination in identifying survivors at elevated likelihood of high-intensity psychosocial engagement.

Supplementary analyses demonstrated that model-predicted risk remained moderately associated with the original non-dichotomized psychosocial service-use score (Supplementary Table S1), suggesting that the risk stratification framework retained meaningful information from the underlying count-based outcome despite dichotomization.

**Fig. 2:**
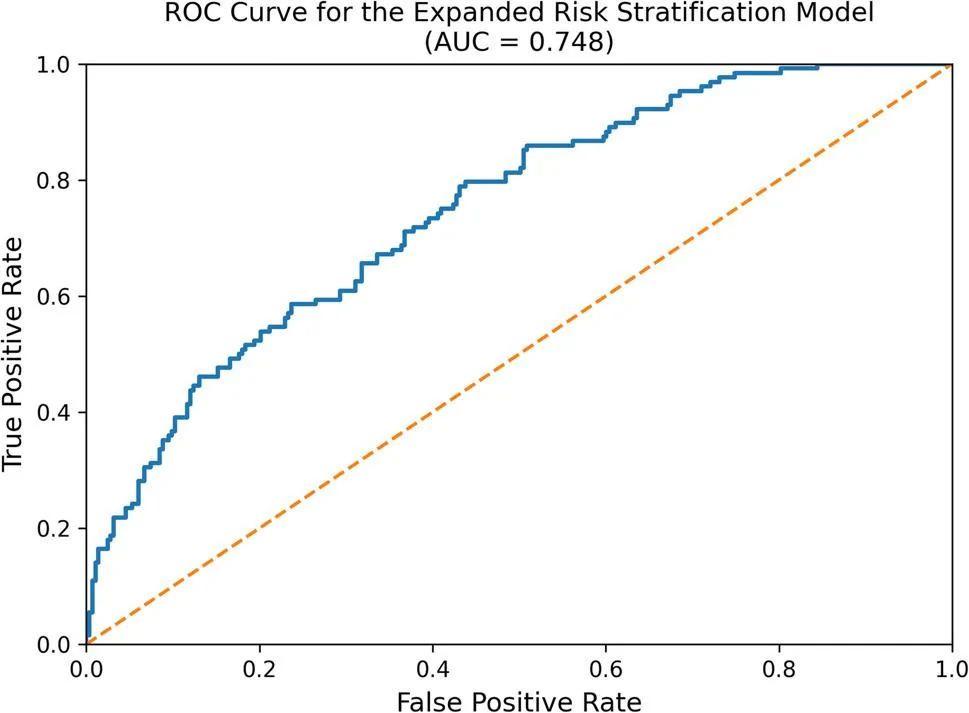
Receiver Operating Characteristic (ROC) Curve for the Expanded Risk Stratification Model Among Women Living with Breast Cancer. Receiver operating characteristic (ROC) curve for the expanded risk stratification model of high-intensity psychosocial service use among women living with breast cancer, including distress burden. The ROC curve illustrates discrimination performance after addition of the distress index to demographic and clinical predictors. The area under the curve (AUC) reflects the model’s ability to correctly classify participants with high-intensity psychosocial service use. AUC = area under the receiver operating characteristic curve. Cross-validated AUC = 0.748. The expanded model includes all base predictors plus the composite distress index. Predicted probabilities were derived using stratified 5-fold cross-validation

**Fig. 3:**
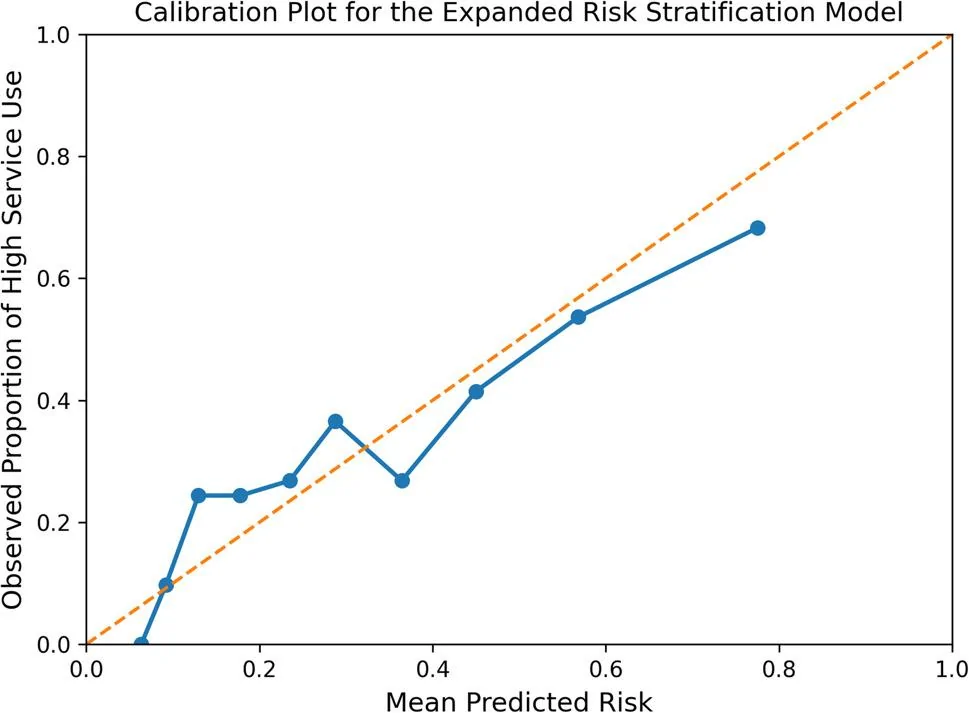
Calibration Plot for the Expanded Risk Stratification Model Among Women Living with Breast Cancer. Calibration plot for the expanded risk stratification model of high-intensity psychosocial service use among women living with breast cancer, including distress burden. Observed versus predicted probabilities of high psychosocial service use across deciles of predicted risk. The diagonal line indicates perfect agreement between observed and predicted outcomes. Calibration assessed using cross-validated predicted probabilities. The expanded model demonstrates improved alignment between predicted and observed risk compared with the base model

Discrimination performance for the expanded model among women living with breast cancer is presented in Fig. 2, and calibration is shown in Fig. 3. The calibration slope and intercept obtained from bootstrap validation were close to their ideal values, indicating good model calibration and acceptable stability.

### Multivariable predictors of high psychosocial service use

Adjusted odds ratios from the expanded logistic regression model are presented in Table 2.

**Table 2:** Multivariable logistic regression predictors of high-intensity psychosocial service use among women living with breast cancer (expanded model)

Predictor	Reference category	OR (95% CI)	*p*-value
Distress index, per unit	—	2.58 (1.90–3.49)	< 0.001
Years since diagnosis, per year	—	0.91 (0.84–0.99)	0.029
Age, per year	—	1.02 (0.99–1.04)	0.195
≥ 1 comorbidity	No comorbidity	0.73 (0.42–1.26)	0.259
Employed	Not employed	0.88 (0.52–1.50)	0.644
Income – Below average	Above average	4.19 (2.21–7.96)	< 0.001
Income – Average	Above average	1.42 (0.73–2.75)	0.302
Income – Prefer not to answer	Above average	6.52 (2.16–19.64)	< 0.001
Metastatic stage	Early/DCIS–III	0.39 (0.13–1.11)	0.078
Survivor/unknown stage	Early/DCIS–III	0.33 (0.19–0.57)	< 0.001

Adjusted odds ratios (OR) and 95% confidence intervals from the expanded multivariable logistic regression model predicting high-intensity psychosocial service use among women living with breast cancer

Conventional (unpenalized) logistic regression estimates are presented to facilitate interpretation of associations between predictor variables and high-intensity psychosocial service use. Penalized regression was used for risk-stratification model development and performance evaluation

Distress index and age were modeled as continuous variables. Years since diagnosis was modeled per additional year since diagnosis. High-intensity psychosocial service use was defined as the upper tertile (17–27) of the cumulative psychosocial service-use score. Income categories were self-reported relative categories referenced to the national average wage

*OR *Odds ratio, *CI *Confidence interval, *DCIS *Ductal carcinoma in situ. Reference categories were above-average income, early-stage disease (DCIS–III), no comorbidity, and not employed

Distress burden was the strongest independent predictor of high psychosocial service use. Lower household income and shorter time since diagnosis were also significantly associated with increased likelihood of high-intensity psychosocial engagement.

### Risk-based triage performance

To evaluate the practical applicability of the proposed framework, we simulated targeted outreach strategies based on predicted risk. The results demonstrated the expected trade-off between outreach efficiency and case identification. More restrictive targeting strategies achieved higher specificity and positive predictive value but identified a smaller proportion of women who subsequently reported high-intensity psychosocial service use.

The addition of distress burden improved performance across all evaluated outreach thresholds. Under a strategy targeting the highest-risk 20% of participants, the expanded model achieved higher sensitivity and positive predictive value than the base model, indicating more efficient concentration of outreach efforts among women most likely to engage intensively with psychosocial support services. Similar improvements were observed under the more restrictive top 10% targeting strategy, where incorporation of distress burden further increased positive predictive value while maintaining high specificity.

Additional implementation analyses demonstrated that broader outreach strategies substantially increased case identification. Targeting the highest-risk 30%, 40%, and 50% of participants captured approximately 52%, 61%, and 73% of high-intensity psychosocial service users, respectively (Supplementary Table S6). Decision curve analysis further demonstrated positive net benefit across clinically relevant threshold probabilities and showed that the expanded model consistently outperformed default non-targeted outreach approaches (Supplementary Figure S3).

Taken together, these findings suggest that the practical utility of the model depends on the outreach capacity and implementation strategy adopted by survivorship programs. While restrictive outreach thresholds maximize efficiency, broader targeting strategies may identify a substantially larger proportion of women likely to require intensive psychosocial support.

Detailed threshold-specific performance metrics are presented in Supplementary Table S2.

## Discussion

In this cross-sectional risk stratification study of breast cancer survivors engaged in community-based psychosocial support services, three findings are salient for survivorship care organization and resource allocation. First, a pragmatic model based on demographic and clinical variables achieved moderate discrimination for identifying high-intensity psychosocial service use. Second, adding a brief composite distress index yielded a meaningful improvement in discrimination and overall classification performance, supporting the practical value of symptom burden information for targeted outreach. Third, risk-based triage metrics suggested that concentrating outreach on the highest predicted-risk strata may increase the efficiency of identifying survivors most likely to engage intensively with psychosocial support services. Taken together, these findings also have relevance for women’s health services, because they identify a feasible approach for prioritizing supportive care among women facing variable levels of psychosocial and socioeconomic burden after breast cancer.

A central challenge in survivorship care is that long-term needs are heterogeneous, evolve over time, and are shaped by factors beyond tumor characteristics. Recent syntheses continue to show that breast cancer survivors commonly report unmet supportive care needs across emotional, informational, and health system domains, with variability by survivorship phase and socioeconomic context [14, 28]. Our findings align with this broader literature by demonstrating that demographic and clinical characteristics provide some signal for high-intensity engagement, but that a brief measure of distress burden substantially enhances the ability to identify those likely to require intensive support.

This result is consistent with contemporary psycho-oncology evidence emphasizing the clinical relevance of distress screening across the cancer journey, while highlighting that screening must be coupled with actionable care pathways to translate into improved support delivery [29, 30]. In practice, survivorship programs often face constrained psychosocial workforce capacity; therefore, the operational question is not only “who is distressed?” but “who is likely to need and use more intensive psychosocial care?” The model developed here addresses this gap by translating routinely collectable information into an individualized probability of high-intensity psychosocial service use. For women’s health practice, this is important because survivorship care frequently extends beyond oncologic follow-up and into broader psychosocial, family, and community contexts in which women may experience layered forms of burden. The findings should be interpreted within both a women’s health and oncology survivorship framework. Breast cancer survivorship is not only an oncologic condition but also a gendered health experience shaped by family roles, employment disruption, body image, reproductive and sexual health concerns, caregiving responsibilities, financial strain, and differential access to supportive care [6]. The observed association between income and high psychosocial service use further suggests that psychosocial engagement may reflect not only distress burden but also socioeconomic vulnerability and help-seeking pathways [31–33].

Risk-stratified survivorship care is increasingly advocated as a way to align survivors with care pathways matching the complexity of their needs. However, much of the survivorship risk-stratification discourse has focused on follow-up intensity, surveillance coordination, and shared care arrangements rather than operational targeting of psychosocial support services specifically [34]. Our study extends this agenda to community-based psychosocial programming by providing a pragmatic risk-stratification framework that can support proactive outreach.

Importantly, the incremental gain from including distress burden speaks directly to implementation decisions. Although the distress index substantially improved model discrimination and classification performance, this finding should be interpreted cautiously. The index has not undergone external validation as an independent diagnostic instrument, and its construct validity outside the present dataset remains uncertain. Consequently, the observed improvement in model performance should be viewed as evidence of pragmatic utility within this setting rather than definitive evidence of broader clinical validity.

Additional exploratory analyses suggested that the distress index was associated with, but not synonymous with, psychosocial service use. Correlations between distress burden and both high-intensity psychosocial service use and the cumulative service-use score were moderate rather than extreme (Supplementary Table S3), indicating related but non-identical constructs. Furthermore, the distress index provided significant incremental classification information beyond demographic and clinical variables, while showing no evidence of problematic collinearity with other predictors (Supplementary Table S3).

Nevertheless, some conceptual overlap may exist between distress burden and psychosocial service engagement. Women experiencing greater emotional distress may be inherently more likely to seek, accept, or be referred to psychosocial support services. Therefore, part of the observed improvement in classification performance may reflect the close relationship between distress burden and service utilization rather than entirely independent information. Future studies should evaluate the incremental value of distress measures using externally validated instruments and independent survivorship cohorts [16, 17].

Notably, the distress index was constructed from brief symptom items embedded within routine survivorship assessment rather than from a full diagnostic instrument, underscoring the feasibility of integrating lightweight screening measures into risk-stratification triage models. Distress screening is widely recommended, yet health systems continue to report gaps in consistent screening and follow-through, particularly as care delivery models evolve [29, 30]. A brief, feasible distress index that materially improves prediction strengthens the case for integrating symptom screening into survivorship workflows when linked to a clear triage mechanism.

From a programmatic perspective, the triage simulations illustrate how a fixed outreach capacity (e.g., contacting the top 10% or 20% of predicted risk) may increase the yield of identifying participants with high psychosocial service use, potentially improving the efficiency of psychosocial outreach and support allocation.

Detailed threshold-specific performance estimates supporting these simulations are provided in Supplementary Table S1. While risk-based targeting must be implemented carefully to avoid excluding individuals with meaningful needs who do not score highly on predicted risk, such approaches may help programs shift from reactive, self-initiated engagement toward proactive identification of survivors likely to require sustained support.

Importantly, the present model was developed to identify women more likely to engage intensively with psychosocial services rather than to directly identify all women with unmet psychosocial needs, and the findings should be interpreted within this context.

Importantly, the performance of the proposed risk-stratification framework should be interpreted in the context of its intended purpose. The objective was not to develop a stand-alone diagnostic tool capable of identifying all women with high psychosocial support needs, but rather to support prioritization of outreach efforts within resource-constrained survivorship programs, consistent with contemporary survivorship risk-stratification frameworks [21, 34]. The expanded model demonstrated favorable overall performance for an internally validated survivorship risk-stratification study, including discrimination in the range of AUC ≈ 0.73–0.75, a Brier score of approximately 0.18, acceptable calibration, bootstrap validation, and cross-validation. In addition, incorporation of distress burden produced meaningful improvements in model performance beyond demographic and clinical predictors. Additional implementation analyses further demonstrated positive net benefit across clinically relevant threshold probabilities (Supplementary Table S6; Supplementary Figure S3), indicating that the model provides useful decision-support information beyond default strategies of contacting all participants or contacting none. Capture-rate analyses also showed that broader outreach strategies identified a substantial proportion of high-intensity service users, with approximately 52%, 61%, and 73% of cases captured when targeting the highest-risk 30%, 40%, and 50% of participants, respectively. Taken together, these findings suggest that the model may improve the efficiency of psychosocial outreach while remaining insufficient as a stand-alone mechanism for identifying all women with psychosocial support needs.

Equity considerations are particularly relevant. Survivorship needs and access to supportive care often vary by socioeconomic status, and recent work continues to emphasize the persistence of unmet supportive care needs in survivorship populations with potential disparities by sociodemographic context [14, 28]. In our model, income-related predictors contributed to risk stratification, suggesting that targeted outreach may help identify survivors who are more likely to engage intensively with psychosocial services in contexts where barriers to navigation and resource access are present. The strong association between lower income and high psychosocial service use is also consistent with the broader literature on financial toxicity in cancer survivorship, whereby economic vulnerability contributes to psychosocial burden, supportive-care needs, and healthcare engagement [31–33]. Additional exploratory analyses demonstrated a significant socioeconomic gradient across income categories, with lower-income women reporting both greater distress burden and higher likelihood of high-intensity psychosocial service use (Supplementary Table S4). Importantly, the association between income and high-intensity service use remained statistically significant after adjustment for distress burden, suggesting that economic vulnerability may influence psychosocial service engagement through multiple pathways beyond emotional distress alone. Future work should explicitly assess model performance across relevant subgroups to ensure that risk-based targeting does not unintentionally amplify inequities. Within a women’s health framework, these findings are especially relevant because financial strain, social roles, and unequal access to supportive services may intensify survivorship challenges for some women more than for others.

Several psychosocial and family-context variables, including marital status, number of children, dependent children, family support, partner support, and friend support, were evaluated during model development and are presented in Supplementary Table S5. Although selected social-support indicators demonstrated statistically significant associations with psychosocial service use, their inclusion produced only modest improvements in discrimination (AUC increase from 0.748 to 0.760) and overall classification performance (Brier score improvement from 0.180 to 0.174). Consistent with recommendations emphasizing model parsimony, interpretability, and transportability, these variables were not retained in the final model because their incremental predictive contribution was limited relative to the additional complexity introduced [21, 35].

This study follows recommended practices for risk stratification model reporting and internal validation. Model development emphasized discrimination and calibration rather than causal inference, consistent with guidance for multivariable model development and validation studies [20]. In addition, contemporary methodological guidance underscores that internal validation is a minimum requirement and that reliance on simple data splitting is discouraged; bootstrap and cross-validation approaches provide more informative internal performance assessment [36, 37]. The use of cross-validated predicted probabilities for both performance estimation and triage simulations improve the interpretability and relevance of results for real-world implementation. An additional strength is the focus on a women-specific survivorship population receiving community-based psychosocial care, which enhances the direct relevance of the findings for women’s supportive health services.

At the same time, because the representativeness of the analytic cohort relative to the broader population served by the support organization could not be formally assessed, external generalizability should be interpreted with appropriate caution.

### Limitations

Several limitations should be considered. First, the analysis is cross-sectional and based on a single community-based support setting; external validation in additional survivorship populations and service contexts is needed before broader deployment. Second, the outcome represents high-intensity psychosocial service use defined by the upper tertile of a cumulative service-use score, which reflects program-specific patterns of service engagement and may require recalibration or threshold adjustment in other settings. The definition of high-intensity psychosocial service use was based on the upper tertile of the program-specific service-use distribution rather than an externally validated clinical threshold. Accordingly, the outcome should be interpreted as a relative measure of psychosocial service-use intensity within this survivorship support setting rather than as a direct measure of clinical psychosocial need. Although this approach supports relative risk stratification within the observed population, the absolute cut-off may not generalize to other survivorship settings. Future studies should evaluate alternative threshold definitions and assess transportability across programs with differing service structures.

An additional limitation relates to the distress index incorporated into the expanded model. Although the index demonstrated excellent internal consistency and a largely unidimensional structure within the present sample, it was derived from seven symptom items embedded within the survivorship survey rather than from a formally validated distress instrument such as the Hospital Anxiety and Depression Scale (HADS), Distress Thermometer, or Generalized Anxiety Disorder scale (GAD-7). Consequently, the distress index should be interpreted as a pragmatic study-specific measure of distress burden rather than a clinically validated diagnostic instrument. Further validation in independent survivorship cohorts is required before broader application.

Although dichotomization may reduce statistical information relative to modelling the original count-based outcome directly [24, 35], a binary outcome was selected to reflect the practical objective of identifying survivors with comparatively high psychosocial service engagement for targeted outreach within a resource-constrained community support setting. To further evaluate the potential impact of this decision, supplementary analyses examined the relationship between model-predicted risk and the original continuous psychosocial service-use score. These analyses demonstrated a moderate association between predicted risk and the underlying non-dichotomized outcome (Supplementary Table S1), suggesting that the risk stratification framework retained meaningful information from the original count-based measure despite dichotomization. Nevertheless, future studies should evaluate whether modelling the original service-use score directly, using approaches such as Poisson or negative binomial regression, provides additional predictive information and improves model performance.

At the same time, supplementary implementation analyses demonstrated positive net benefit across a broad range of threshold probabilities and substantial capture of high-intensity service users at more inclusive outreach thresholds (Supplementary Table S6; Supplementary Figure S3), suggesting that the model may still provide useful support for prioritization decisions despite its imperfect sensitivity.

Several limitations are particularly relevant to interpretation. First, the study relied on cross-sectional self-reported survey data, and therefore the model should be interpreted as a concurrent classification model rather than a prognostic prediction model [24]. Second, the outcome reflects observed psychosocial service use within a service-engaged population and should not be interpreted as equivalent to unmet psychosocial need. Service use may be influenced by awareness, accessibility, referral pathways, help-seeking preferences, social support, and structural barriers rather than psychosocial need alone [6, 17]. Accordingly, high psychosocial service utilization should not be interpreted as synonymous with unmet psychosocial need. Some women with substantial psychosocial needs may never engage with available services, whereas others may demonstrate relatively high utilization despite lower levels of unmet need. Because the analytic sample consisted of women who completed a survey disseminated through a survivorship support organization and related community/professional channels, the model may preferentially identify correlates of service engagement or help-seeking behavior rather than women with the greatest unmet psychosocial need. This introduces a potential risk of selection and help-seeking bias and limits the extent to which the findings can be generalized to breast cancer survivors who are less aware of, less connected to, or unable to access psychosocial support services. Although supplementary analyses demonstrated a significant socioeconomic gradient in both distress burden and psychosocial service use together with an independent association between income and high-intensity psychosocial service use after adjustment for distress burden (Supplementary Table S4), these findings should not be interpreted as evidence that income directly reflects psychosocial need. Differences in awareness, access to services, referral pathways, and help-seeking behavior may also contribute to the observed associations.

In addition, although the distress index demonstrated excellent internal consistency and a largely unidimensional structure within the present sample, it was derived from seven symptom items embedded within the survivorship survey rather than from a formally validated distress instrument such as the Hospital Anxiety and Depression Scale (HADS), Distress Thermometer, or Generalized Anxiety Disorder scale (GAD-7). Consequently, the distress index should be interpreted as a pragmatic study-specific measure of distress burden rather than a clinically validated diagnostic instrument, and further validation in independent survivorship cohorts is warranted. Third, despite the incremental predictive contribution of the distress index beyond demographic and clinical predictors and the absence of problematic collinearity (Supplementary Table S3), some conceptual overlap between distress burden and psychosocial service engagement may remain. Because emotional distress may influence help-seeking behavior, referral patterns, and engagement with psychosocial services, part of the observed improvement in classification performance may reflect this relationship. Future studies should evaluate the incremental value of distress measures using externally validated instruments and independent survivorship cohorts [16, 17].

Several psychosocial and family-context variables were evaluated during model development (Supplementary Table S5). Although selected social-support indicators demonstrated statistically significant associations with psychosocial service use, their inclusion resulted in only modest improvements in discrimination and overall classification performance. Future studies should evaluate whether validated measures of social support, caregiving burden, family functioning, and relationship quality improve model performance and transportability across survivorship settings.

Fourth, predictors were based on self-reported survey measures and categorized clinical information; misclassification is possible, and future studies could assess whether integration of clinical registry variables improves model performance. In addition, although the study is directly relevant to women’s health, the sample was drawn from one national support organization in Israel, and the findings may not fully capture the diversity of women’s survivorship experiences across healthcare systems, cultures, or underserved subpopulations. In addition, because recruitment occurred through multiple community-based, online, and professional dissemination channels, the total number of women exposed to the survey invitation could not be determined and a formal survey response rate could not be calculated.

Furthermore, because detailed demographic and clinical information describing the full population served by the support organization was not available, formal evaluation of sample representativeness was not possible. Participants who chose to complete the survey may have differed from non-participants in psychosocial engagement, awareness of support services, health literacy, digital access, motivation to participate in survivorship-related research, or willingness to seek psychosocial support. Consequently, the extent to which the analytic cohort reflects the broader population of women served by the organization remains uncertain, and the generalizability of the findings should be interpreted cautiously.

### Future directions

Future research should externally validate the model in independent survivorship cohorts, evaluate transportability across community- and oncology-based settings, assess whether modelling the original count-based psychosocial service-use outcome directly (e.g., Poisson or negative binomial approaches) improves performance compared with dichotomized risk-stratification models, examine calibration and decision-analytic utility across implementation settings, determine whether model performance differs across socioeconomic, age, family-status, minority-status, and comorbidity subgroups, further investigate the mechanisms linking socioeconomic vulnerability, distress burden, and psychosocial service engagement, including the potential roles of financial toxicity, access barriers, and help-seeking behavior and evaluate whether social-support resources, family structure, caregiving responsibilities, and relationship factors provide meaningful incremental value beyond distress burden and traditional clinical predictors. Such work will help establish the generalizability, equity implications, and practical usefulness of risk-stratified psychosocial outreach strategies.

## Conclusions

In summary, a pragmatic risk stratification model using routinely collectable demographic and clinical predictors demonstrated moderate ability to identify breast cancer survivors likely to engage in high-intensity psychosocial service use. Incorporating a brief distress index meaningfully enhanced model discrimination and classification performance and increased the efficiency of simulated risk-based outreach. These findings support the potential value of a risk stratification approach for community-based psychosocial survivorship services and suggest that such an approach may assist proactive care planning and resource allocation within supportive cancer care systems. The central implication of these findings is that risk-stratified outreach may assist women-centered services in prioritizing supportive care efforts among breast cancer survivors who may be at elevated risk of intensive psychosocial service use, potentially improving efficiency and equity in outreach activities. Additional implementation analyses demonstrated positive net benefit across clinically relevant threshold probabilities and substantial capture of high-intensity psychosocial service users at broader outreach thresholds. These findings suggest that the proposed framework may provide useful decision-support information for prioritizing psychosocial outreach and resource allocation within survivorship care programs, particularly when implemented as a complement to existing psychosocial assessment processes. However, the model should be viewed as a pragmatic risk-stratification tool intended to support, rather than replace, comprehensive psychosocial assessment and clinical judgment.

## Supplementary Information

Supplementary Material 1.

Supplementary Material 2: Supplementary Figure S1. Receiver Operating Characteristic (ROC) Curve for the Base Risk Stratification Model Among Women Living with Breast Cancer. Receiver operating characteristic (ROC) curve for the base risk stratification model of high-intensity psychosocial service use among women living with breast cancer, including demographic and clinical predictors only. The ROC curve displays the relationship between sensitivity and 1-specificity across all possible probability thresholds using 5-fold cross-validated predicted probabilities. The diagonal reference line represents discrimination equivalent to chance. The area under the curve (AUC) quantifies the model’s ability to distinguish between participants with high versus lower psychosocial service use. Notes: Cross-validated AUC = 0.675. Bootstrap median AUC = 0.706 (95% CI 0.687–0.715). The base model included age, years since diagnosis, employment status, income category, comorbidity status, and grouped disease stage. Predicted probabilities were generated using stratified 5-fold cross-validation.

Supplementary Material 3: Supplementary Figure S2. Calibration Plot for the Base Risk Stratification Model Among Women Living with Breast Cancer. Calibration plot for the base risk stratification of high-intensity psychosocial service use among women living with breast cancer across deciles of predicted risk. Observed proportions of high psychosocial service use are plotted against mean predicted probabilities within deciles of risk. The 45-degree line indicates perfect calibration. Points represent decile-specific observed versus predicted risk. Calibration was assessed using cross-validated predicted probabilities. Bootstrap internal validation (500 resamples) yielded: • Calibration slope ≈ 0.89. • Calibration intercept ≈ -0.07. These values suggest acceptable internal calibration with limited overfitting.

Supplementary Material 4: Supplementary Figure S3. Decision Curve Analysis for the Expanded Risk-Stratification Model. Decision curve analysis comparing the expanded risk-stratification model with default strategies of contacting all participants or contacting none. Positive net benefit across a range of threshold probabilities indicates that the model may provide useful decision-support information for prioritizing psychosocial outreach among breast cancer survivors. The expanded model demonstrated greater net benefit than the treat-all strategy across most clinically relevant threshold probabilities.

## Data Availability

The datasets generated and/or analyzed during the current study are available from the corresponding author on reasonable request, subject to confidentiality protections required by the ethics committee.

## Abbreviations

AUC: Area under the receiver operating characteristic curve
CI: Confidence interval
DCA: Decision curve analysis
DCIS: Ductal carcinoma in situ
EPV: Events per variable
NPV: Negative predictive value
OR: Odds ratio
PPV: Positive predictive value
ROC: Receiver operating characteristic
SD: Standard deviation
TRIPOD: Transparent reporting of a multivariable prediction model for individual prognosis or diagnosis
