# Supplementary material for: Development and internal validation of a risk stratification model for high-intensity psychosocial service use among women living with and beyond breast cancer
Source: BMC Womens Health. 2026 Jul 30;26:558. doi: 10.1186/s12905-026-04726-2 (PMC13711876; doi:10.1186/s12905-026-04726-2)
Supplement: Supplementary file 1 [file 12905_2026_4726_MOESM1_ESM.docx]

**Supplementary Material**

**Risk Stratification for High-Intensity Psychosocial Service Use in Breast Cancer Survivorship**

**Supplementary Figure S1**

**Receiver Operating Characteristic (ROC) Curve for the Base Risk Stratification Model Among Women Living with Breast Cancer**

**Caption:**
Receiver operating characteristic (ROC) curve for the base risk stratification model of high-intensity psychosocial service use among women living with breast cancer, including demographic and clinical predictors only.

**Description:**
The ROC curve illustrates discrimination performance using 5-fold stratified cross-validated predicted probabilities. The curve displays sensitivity versus 1-specificity across all classification thresholds. The diagonal reference line represents performance equivalent to chance discrimination.

**Notes:**
Cross-validated AUC = 0.675.

Bootstrap median AUC = 0.706 (95% CI 0.687–0.715).

The base model included age, years since diagnosis, employment status, income category, comorbidity status, and grouped disease stage. Predicted probabilities were generated using stratified 5-fold cross-validation.

**Supplementary Figure S2**

**Calibration Plot for the Base Risk Stratification Model Among Women Living with Breast Cancer**

**Caption:**
Calibration plot for the base risk stratification model of high-intensity psychosocial service use among women living with breast cancer across deciles of estimated risk.

**Description:**
Observed proportions of high-intensity psychosocial service use are plotted against mean predicted probabilities within deciles of predicted risk. The 45-degree reference line indicates perfect calibration. Points represent decile-specific observed versus predicted risk estimates.

**Notes:**
Calibration was assessed using cross-validated predicted probabilities.

Bootstrap internal validation (500 resamples) yielded:

- Calibration slope ≈ 0.89
- Calibration intercept ≈ -0.07

These values suggest acceptable internal calibration with limited overfitting.

**Supplementary Figure S3**

**Decision Curve Analysis for the Expanded Risk-Stratification Model**

**Caption:**
Decision curve analysis comparing the expanded risk-stratification model with default strategies of contacting all participants or contacting none. Positive net benefit across a range of threshold probabilities indicates that the model may provide useful decision-support information for prioritizing psychosocial outreach among breast cancer survivors. The expanded model demonstrated greater net benefit than the treat-all strategy across most clinically relevant threshold probabilities.

**Supplementary Table S1. Distribution of the Original Psychosocial Service-Use Score and Association with Predicted Risk**

**Caption:**
Distribution of the original psychosocial service-use score and association between predicted risk from the expanded risk stratification model and the underlying non-dichotomized psychosocial service-use outcome among women living with breast cancer.

**Description:**
This table summarizes the distribution of the original psychosocial service-use score (range 0–27) and evaluates whether predicted probabilities generated by the expanded risk stratification model remained associated with the underlying continuous outcome despite dichotomization for model development.

| **Analysis** | **Result** |
| --- | --- |
| Number of participants | 411 |
| Mean psychosocial service-use score | 13.19 |
| Standard deviation (SD) | 6.76 |
| Median | 12 |
| First quartile (Q1) | 8 |
| Third quartile (Q3) | 18 |
| Interquartile range (IQR) | 8–18 |
| Minimum | 0 |
| Maximum | 27 |
| Spearman correlation between predicted risk and original psychosocial service-use score | r = 0.446, p < 0.001 |

**Notes:**
The original psychosocial service-use outcome was a cumulative count score ranging from 0 to 27, with higher values indicating broader engagement with psychosocial support services. High-intensity psychosocial service use in the primary analysis was defined as the upper tertile of the original score (17–27). Predicted risk was derived from the expanded cross-validated risk stratification model that included demographic, clinical, and distress-burden variables. The Spearman correlation analysis was conducted to assess whether model-predicted risk remained associated with the underlying continuous psychosocial service-use outcome after dichotomization. The observed moderate correlation suggests that the risk stratification model retained meaningful information from the original count-based outcome despite the use of a binary classification framework.

**Supplementary Table S2. Risk-Based Triage Performance Metrics for Predicting High-Intensity Psychosocial Service Use Among Women Living with Breast Cancer**

**Caption:**
Sensitivity, specificity, positive predictive value (PPV), and negative predictive value (NPV) under simulated outreach strategies targeting the highest estimated-risk strata among women living with breast cancer.

**Description:**
Performance metrics calculated using cross-validated predicted probabilities for both the base and expanded models. Outreach thresholds were defined as the top 20% and top 10% of predicted risk.

| **Model** | **Threshold** | **Sensitivity** | **Specificity** | **PPV** | **NPV** |
| --- | --- | --- | --- | --- | --- |
| Base | Top 20% | 0.328 | 0.855 | 0.506 | **0.738** |
| Base | Top 10% | 0.195 | 0.940 | 0.595 | **0.721** |
| Expanded | Top 20% | 0.391 | 0.883 | 0.602 | **0.762** |
| Expanded | Top 10% | 0.227 | 0.954 | 0.690 | **0.732** |

**Notes:**
PPV = positive predictive value; NPV = negative predictive value.

High-intensity service use was defined as the upper tertile (17–27) of the cumulative psychosocial service-use score.

Metrics derived from out-of-fold predicted probabilities to avoid optimistic bias.

**Supplementary Methods Note**

Internal validation followed TRIPOD recommendations for multivariable model development and validation. Discrimination was assessed using 5-fold stratified cross-validation. Calibration was evaluated using decile-based calibration plots and bootstrap resampling (500 iterations) to estimate optimism-corrected performance metrics, including calibration slope and intercept. Ridge-penalized logistic regression (L2 regularization) was applied to improve coefficient stability and reduce overfitting.

**Supplementary Table S3. Additional Assessment of the Distress Index and Its Relationship with Psychosocial Service Use**

**Caption:**
Exploratory analyses examining the relationship between the composite distress index and psychosocial service use among women living with breast cancer.

**Description:**
The table presents additional analyses conducted to assess whether the distress index contributed incremental classification information beyond demographic and clinical variables and whether its association with psychosocial service use suggested problematic overlap with the study outcome.

| **Analysis** | **Result** | **Interpretation** |
| --- | --- | --- |
| Point-biserial correlation with high-intensity psychosocial service use | r = 0.357, p < 0.001 | Moderate association; not complete overlap |
| Spearman correlation with cumulative psychosocial service-use score | r = 0.407, p < 0.001 | Moderate monotonic association |
| Likelihood-ratio test: base model vs. expanded model | χ²(1) = 42.09, p < 0.001 | Significant incremental contribution beyond demographic and clinical variables |
| Variance inflation factor (VIF) for distress index in the expanded model | 1.09 | No evidence of problematic collinearity |

**Notes:**
The distress index was calculated as the respondent-level mean of seven psychological symptom items scored from 0 to 3, with higher values indicating greater distress burden. High-intensity psychosocial service use was defined as the upper tertile of the cumulative psychosocial service-use score (17–27). Correlation analyses were based on participants with non-missing distress-index data (n = 401). The likelihood-ratio test compared the base logistic regression model including demographic and clinical variables with the expanded model additionally including the distress index. VIF = variance inflation factor.

**Supplementary Table S4. Additional Analyses of Income, Distress Burden, and High-Intensity Psychosocial Service Use**

**Caption:**

Additional analyses evaluating the relationship between socioeconomic position, distress burden, and high-intensity psychosocial service use among women living with breast cancer.

**Description:**

Exploratory analyses were conducted to examine whether income-related differences in psychosocial service use reflected a broader socioeconomic gradient and whether distress burden contributed to the observed associations. Income categories were analyzed as an ordinal variable (below average, average, above average). Associations with distress burden and high-intensity psychosocial service use were evaluated using correlation and logistic regression analyses.

| **Analysis** | **Result** |
| --- | --- |
| Ordinal income trend predicting high-intensity psychosocial service use | β = −0.760, p < 0.001 |
| Mean distress score – Below-average income | 1.448 |
| Mean distress score – Average income | 1.295 |
| Mean distress score – Above-average income | 1.026 |
| Spearman correlation between income level and distress burden | ρ = −0.201, p < 0.001 |
| Adjusted association between income level and high-intensity psychosocial service use (controlling for distress burden) | β = −0.651, p < 0.001 |
| Association between distress burden and high-intensity psychosocial service use in the adjusted model | β = 0.871, p < 0.001 |

**Notes:**

Income level was modeled as an ordinal variable coded as 1 = below-average income, 2 = average income, and 3 = above-average income. Distress burden was calculated as the respondent-level mean of the seven distress items included in the composite distress index, with higher values indicating greater distress burden. Logistic regression coefficients (β) are reported on the log-odds scale. Negative coefficients indicate lower odds of high-intensity psychosocial service use with increasing income level. High-intensity psychosocial service use was defined as the upper tertile (17–27) of the cumulative psychosocial service-use score.

**Supplementary Table S5. Exploratory Analyses of Psychosocial and Family Context Variables and High-Intensity Psychosocial Service Use**

**Caption:**

Exploratory associations between psychosocial and family-context variables and high-intensity psychosocial service use among women living with breast cancer.

**Description:**

Additional analyses were conducted in response to reviewer comments to evaluate whether psychosocial and family-context variables not included in the primary risk-stratification model were associated with high-intensity psychosocial service use. Univariable associations were examined, followed by assessment of selected variables within an expanded model that additionally adjusted for distress burden and income level. Changes in model discrimination and overall classification performance were also evaluated.

| **Variable** | **Univariable Association with High-Intensity Service Use** | **p-value** | **Retained Statistical Significance After Adjustment for Distress and Income** |
| --- | --- | --- | --- |
| Marital status | Not significant | 0.350 | No |
| Number of children | Not significant | 0.233 | No |
| Number of dependent children | Significant | 0.041 | Yes |
| Need for family support | Not significant | 0.165 | No |
| Need for partner support | Significant | <0.001 | Yes |
| Received family support | Significant | <0.001 | No |
| Received partner support | Significant | <0.001 | No |
| Friend support | Significant | <0.001 | No |

**Expanded Model Performance**

| **Model** | **AUC** | **Brier Score** |
| --- | --- | --- |
| Primary risk-stratification model | 0.748 | 0.180 |
| Expanded model including psychosocial and family-context variables | 0.741 | 0.183 |
| Change in performance | -0.007 | +0.003 |

**Notes:**

High-intensity psychosocial service use was defined as the upper tertile (17–27) of the cumulative psychosocial service-use score. Univariable associations were evaluated using logistic regression models. The expanded model additionally included marital status, number of children, number of dependent children, perceived family support, perceived partner support, and friend support, together with the predictors included in the primary model. Adjusted significance was assessed after controlling for distress burden and income level. Model discrimination was evaluated using the area under the receiver operating characteristic curve (AUC), and overall classification performance was summarized using the Brier score.

**Supplementary Table S6**

**Caption**

**Supplementary Table S6. Additional Evaluation of the Practical Utility of the Expanded Risk-Stratification Model**

**Description**

To further evaluate the implementation utility of the expanded risk-stratification model, additional analyses were conducted beyond conventional discrimination and calibration measures. Panel A presents capture-rate analyses across alternative outreach thresholds, illustrating the proportion of high-intensity psychosocial service users identified when outreach resources are targeted to progressively larger high-risk strata. Panel B presents decision curve analysis (DCA) results, comparing the net benefit of the expanded model with default strategies of contacting all participants or contacting none. Together, these analyses provide insight into the potential practical value of the model for prioritizing psychosocial outreach in survivorship care settings.

**Panel A. Capture-Rate Analysis**

| **Outreach Threshold** | **Population Contacted n (%)** | **High-Intensity Users Captured n (%)** | **Sensitivity** | **PPV** | **NPV** |
| --- | --- | --- | --- | --- | --- |
| Top 10% | 42 (10.2%) | 29 (22.7%) | 0.227 | 0.690 | 0.732 |
| Top 20% | 83 (20.2%) | 50 (39.1%) | 0.391 | 0.602 | 0.762 |
| Top 30% | 124 (30.2%) | 67 (52.3%) | 0.523 | 0.540 | 0.787 |
| Top 40% | 165 (40.1%) | 78 (60.9%) | 0.609 | 0.473 | 0.797 |
| Top 50% | 206 (50.1%) | 94 (73.4%) | 0.734 | 0.456 | 0.834 |

**Panel B. Decision Curve Analysis**

| **Threshold Probability** | **Expanded Model Net Benefit** | **Treat-All Strategy** | **Treat-None Strategy** |
| --- | --- | --- | --- |
| 0.05 | 0.2765 | 0.2752 | 0.0000 |
| 0.10 | 0.2474 | 0.2349 | 0.0000 |
| 0.15 | 0.2035 | 0.1899 | 0.0000 |
| 0.20 | 0.1782 | 0.1393 | 0.0000 |
| 0.25 | 0.1395 | 0.0819 | 0.0000 |
| 0.30 | 0.1057 | 0.0163 | 0.0000 |
| 0.35 | 0.0827 | -0.0593 | 0.0000 |
| 0.40 | 0.0722 | -0.1476 | 0.0000 |
| 0.45 | 0.0619 | -0.2519 | 0.0000 |
| 0.50 | 0.0438 | -0.3771 | 0.0000 |
| 0.55 | 0.0308 | -0.5301 | 0.0000 |
| 0.60 | 0.0231 | -0.7214 | 0.0000 |

**Note:**

High-intensity psychosocial service use was defined as the upper tertile of the cumulative psychosocial service-use score (utilization score ≥17). Capture-rate analyses were based on cross-validated predicted probabilities generated by the expanded risk-stratification model. Sensitivity represents the proportion of high-intensity service users correctly identified within each targeted outreach stratum. PPV represents the probability that a participant targeted for outreach belongs to the high-intensity service-use group, whereas NPV represents the probability that a participant not targeted for outreach does not belong to the high-intensity service-use group.

Decision curve analysis evaluated the clinical and operational utility of the expanded model across a range of threshold probabilities. Positive net benefit values indicate that risk-based outreach using the model provides greater benefit than default strategies of contacting all participants or contacting none. Across all evaluated thresholds, the expanded model demonstrated positive net benefit and outperformed the treat-all strategy, supporting its potential usefulness as a decision-support tool for prioritizing psychosocial outreach in survivorship care settings.

**Supplementary Appendix A**

**Items Included in the Composite Distress Index**

**Table A1. Survey Items Included in the Composite Distress Index**

| **Item** | **Survey Question** |
| --- | --- |
| 1 | Felt nervous, anxious, or very tense |
| 2 | Unable to stop or control worrying |
| 3 | Worried too much about different things |
| 4 | Had difficulty relaxing |
| 5 | Was so restless that it was difficult to sit still |
| 6 | Became easily annoyed or irritable |
| 7 | Felt afraid as if something awful might happen |

**Note.** All items were scored using a four-category response scale:

- 0 = Not at all
- 1 = Several days
- 2 = More than half the days
- 3 = Nearly every day

The composite distress index was calculated as the arithmetic mean of the seven items (range 0–3), with higher scores indicating greater psychological distress.
